# SARS-CoV-2 infection in kidney transplant recipients: clinical impact and outcomes - a single center experience

**DOI:** 10.1590/2175-8239-JBN-2021-0164

**Published:** 2021-11-19

**Authors:** Afonso Santos, Luís Leite de Sousa, Rita Calça, Anna Lima, Célia Nascimento, Cristina Jorge, Teresa Adragão, Margarida Bruges, Susana Peres, André Weigert

**Affiliations:** 1 Hospital Professor Fernando da Fonseca, Departamento de Nefrologia, Amadora, Portugal.; 2 Hospital de Santa Cruz, Centro Hospitalar de Lisboa Ocidental, Departamento de Nefrologia, Lisboa, Portugal.; 3 Hospital Egas Moniz, Centro Hospitalar de Lisboa Ocidental, Departamento de Doenças Infecciosas, Lisboa, Portugal.

**Keywords:** Kidney Transplantation, COVID-19, Antivirals, Transplante de Rim, COVID-19, Antivirals

## Abstract

**Introduction:**

Kidney transplant recipients are a subgroup of patients at higher risk of critical forms of Severe Acute Respiratory Syndrome Coronavirus-2 (SARS-CoV-2) infection and poor outcomes due to immunosuppression treatment. Herein, we present data from a single center cohort of kidney transplant recipients with SARS-CoV-2 infection.

**Methods:**

In a prospective study, baseline characteristics, clinical features, antiviral and immunosuppression management were compared between outpatients and hospitalized patients, during a one-year period.

**Results:**

Seventy-seven kidney transplant recipients were analyzed, including outpatients and hospitalized patients, with a median age of 57.7 (IQR 49.7-64.9) years. Twenty-eight (36.4%) were managed as outpatients, while 49 (63.6%) patients required hospital admission. Among hospitalized patients, 18.4% were admitted in ICU, 49% had AKI, and 20.4% died. Immunosuppression adjustments were performed in 95.9% of hospitalized patients, with dose of anti-metabolites adjusted in 83.7%, mTOR inhibitors in 14.3%, calcineurin inhibitors in 12.2%, and corticosteroid therapy in 81.6%.

**Conclusion:**

Among hospitalized patients, immunosuppression management included reduction or withdrawal of anti-metabolite and increase of corticosteroid dose. AKI occurred in almost half of patients and mortality in hospitalized patients reached 20%, reflecting greater disease severity than the general population.

## RESUMO

**Introdução:**

Receptores de transplante renal são um subgrupo de doentes com maior risco de apresentar formas críticas de infecção por Síndrome Respiratória Aguda Grave pelo Coronavirus-2 (SARS-CoV-2) e piores *outcomes* devido ao tratamento imunossupressor. Aqui, apresentamos dados de uma coorte de um único centro de receptores de transplante renal com infecção por SARS-CoV-2.

**Métodos:**

Num estudo prospectivo, características basais, características clínicas, adaptação da terapêutica antiviral e de imunossupressão foram comparados entre doentes seguidos em ambulatório e doentes hospitalizados durante um período de um ano.

**Resultados:**

Foram analisados setenta e sete receptores de transplante renal, incluindo doentes de ambulatório e hospitalizados, com idade média de 57,7 (IIQ 49,7-64,9) anos. Vinte e oito (36,4%) foram tratados em ambulatório enquanto 49 (63,6%) doentes necessitaram de internação hospitalar. Entre os doentes hospitalizados, 18,4% foram admitidos na UTI, 49% apresentaram LRA, e 20,4% morreram. Foram realizados ajustes de imunossupressão em 95,9% dos pacientes hospitalizados, com dose de antimetabólitos ajustada em 83,7%, inibidores de mTOR em 14,3%, inibidores de calcineurina em 12,2%, e terapia com corticosteroides em 81,6%.

**Conclusão:**

Entre os pacientes hospitalizados, a optimização da terapêutica imunossupressora incluiu redução ou retirada de antimetabólito e aumento da dose de corticosteroides. A LRA ocorreu em quase metade dos pacientes e a mortalidade em pacientes hospitalizados atingiu 20%, refletindo uma maior gravidade da doença em relação à população em geral.

## Introduction

The SARS-CoV-2 infection and coronavirus disease 2019 (COVID-19) have spread among the global population since March 2020, and many reports of their clinical impact have been published since then. Moreover, there has been increased clinical interest on its effects in terms of presentation, severity and disease management in certain subgroups of patients, such as transplant recipients.

In this regard, many countries and transplant societies published their own recommendations in early 2020, and consensus documents and guidelines have subsequently emerged^1^^,^ 2. However, the exact clinical impact of SARS-CoV-2 infection in kidney transplant recipients is still unknown and remains a subject of interest. The pandemic continues to evolve and by April 2021 more than 127 million people have been infected and almost 3 million have died due to COVID-19, according to the European Centre for Disease Prevention and Control (ECDC) data^3^.

Solid organ transplant recipients have been described as particularly at risk for developing severe COVID-19, and various reports from Italy, Spain, France, and United States showed mortality rates ranging from 25 to 28% of infected patients^4^^-^ 7. In Portugal, as in many other countries around the world, the pandemic had a major impact on the national health care system and resulted in high mortality rates. This impact was also seen among kidney transplant recipients.

The aim of our report was to present the clinical impact of COVID-19 in a cohort of kidney transplant recipients followed in a single transplantation center in Lisbon. We present the main clinical features of patients diagnosed with SARS-CoV-2 infection.

## Methods

A single center observational and prospective cohort study was performed. All kidney transplant recipients followed at Kidney Transplantation Centre (KTC) at Hospital de Santa Cruz in Lisbon were considered for inclusion in the study. We included those diagnosed with SARS-CoV-2 infection through a naso- and/or oropharyngeal swab and real time-polymerase chain reaction test (RT-PCR) between March 1^st^ 2020 and February 28^th^ 2021.

Clinical records were manually reviewed for demographics, immunosuppression changes, and laboratory findings. Acute kidney injury (AKI) was graded according to Kidney Disease Improving Global Outcomes (KDIGO) criteria.

Descriptive analysis was performed using the IBM^®^ SPSS Statistics software for Windows, version 22. Categorical variables are presented as frequency distributions or percentages. Continuous variables are presented as median and interquartile range (IQR) [25th-75th percentile (P25 to P75)]. Due to the small sample size, non-parametric tests were used. For continuous variables, the comparison of medians was performed with Mann-Whitney/related samples Wilcoxon signed rank. For all comparisons and correlations a probability value <0.05 was considered significant.

The study had the approval of the Ethical Committee of the institution.

## Results

Of the approximately 950 kidney transplant recipients seen at our center in 2020, 77 patients were diagnosed with SARS-CoV-2 infection between March 2020 and February 2021. Of these, 28 (36.4%) were treated as outpatients, while 49 (63.6%) were hospitalized at some point of their disease.

## Patients’ characteristics

At the time of diagnosis of SARS-CoV-2 infection, the median age of patients was 57.7 (IQR 49.7 - 64.9) years. Most patients (53.2%) were male, 85.7% were Caucasian, and 14.3% were Black (Table 1).

**Table 1:** Baseline demographics, comorbidities, and mortality of outpatients and hospitalized kidney transplant recipients with COVID-19

	Total (n =77)	Outpatients (n=28)	Inpatients (n=49)	*p*-value
Age (years)	57.7 (IQR 49.7 - 64.9)	53.9	58.3	0.812
Male (%)	53.2 (n=41)	53.6 (n=15)	53.1 (n=26)	0.966
Black race (%)	14.3 (n=11)	17.9 (n=5)	12.2 (n=6)	0.498
Comorbidities (%)				
Hypertension	87 (n=67)	85.7 (n=24)	87.8 (n=43)	0.798
*Diabetes mellitus*	28.6 (n=22)	7.1 (n=2)	40.8 (n=20)	0.002
Dyslipidemia	39 (n=30)	25 (n=7)	46.9 (n=23)	0.058
Cardiovascular disease	15.6 (n=12)	7.1 (n=2)	20.4 (n=10)	0.123
Overweight	35.1 (n=27)	28.6 (n=8)	36.7 (n=18)	0.466
Obesity	13 (n=10)	7.1 (n=2)	18.4 (n=9)	0.176
BMI ≥ 25 kg/m^2^	48.1 (n=37)	35.7 (n=10)	55.1 (n=27)	0.101
Time from transplant to COVID-19 diagnosis (years)	9.3 (IQR 3.8 – 12.3)	9.7 (IQR 5.5-14.2)	7.0 (IQR 2.8-12.1)	0.234
Deceased donor (%)	87 (n=67)	85.7 (n=24)	87.7 (n=43)	0.798
Mortality (%)	13 (n=10)	0% (n=0)	20.4 (n=10)	0.010

BMI: Body mass index.

Hypertension was the most common comorbidity, affecting 87% of patients, followed by dyslipidemia (39%), diabetes (28.6%), overweight (35.1%), obesity (13%), and 15.6% had known cardiovascular disease.

Median time to diagnosis of COVID-19 after transplantation was 9.3 years (IQR 3.8-12.3), and 10.4% (n=8) of patients had been transplanted in the previous six months. The majority of patients included in this analysis had undergone a deceased kidney transplant (87%).

## Clinical outcomes

Of the hospitalized patients (n=49), 18.4% were admitted to Intensive Care Unit (ICU) and the remaining 81.6% were treated in the ward (Table 2). Mean time of hospitalization, including patients who did not survive, was 17 (IQR 9-27) days.

**Table 2:** Baseline demographics and comorbidities of hospitalized patients: survivors vs. non-survivors

	Inpatients (n=49)		
	Survivors (n =39)	Non-survivors (n=10)	*p*-value
Age (years)	57 (IQR 51.1-64.4)	65 (IQR 58.3-70.6)	0.047
Male (%)	53.8 (n=21)	50 (n=5)	0.828
Black race (%)	7.7 (n=3)	30 (n=3)	0.055
Comorbidities (%)			
Hypertension	84.6 (n=33)	100 (n=10)	0.185
*Diabetes mellitus*	38.5 (n=15)	50 (n=5)	0.508
Dyslipidemia	41 (n=16)	70 (n=7)	0.101
Cardiovascular disease	20.5 (n=8)	20 (n=2)	0.971
Overweight	35.9 (n=14)	40 (n=4)	0.810
Obesity	20.5 (n=8)	10 (n=1)	0.444
BMI ≥ 25 kg/m^2^	56.4 (n=22)	50 (n=5)	0.716
Time from transplant to COVID-19 diagnosis (years)	7 (IQR 2.5-12.2)	8 (IQR 5.0-10.2)	0.893
Deceased donor (%)	84.6 (n=33)	100 (n=10)	0.185

BMI: Body mass index.

Almost all hospitalized patients (95.9%) had their immunosuppression adjusted after diagnosis. The dose of anti-metabolite (mycophenolate mofetil, mycophenolic acid, or azathioprine) were reduced or discontinued in 83.7%, mammalian target of rapamycin inhibitors (everolimus or sirolimus) were reduced or discontinued in 14.3%, and calcineurin inhibitors (tacrolimus or cyclosporine) were either reduced or discontinued in 12.2% of patients. Corticosteroid therapy was intensified in the majority of patients (81.6%).

For COVID-19 treatment, the most frequently used medication was dexamethasone, in 57.1% of patients, followed by remdesivir, used in 38.8% of patients; 34.7% of patients received both treatments. In most cases, treatment with remdesivir included 200 mg in the first day, followed by 100 mg in the next four days.

AKI occurred in 49% of patients, with a median maximum serum creatinine of 2.27 (IQR 1.6-4.1) mg/dL, KDIGO 1 in 50% (n=12), KDIGO 2 in 12.5% (n=3), and KDIGO 3 in 20.8% (n=5). Kidney replacement therapy (KTR) was required in 12.2% of cases and mortality occurred in 20.4%.

## Outpatients vs. inpa tients

There were no significant differences among patients with COVID-19 treated as outpatients *versus* patients who needed hospital admission. The only exception was the relative frequency of diabetes mellitus in the latter group, which was significantly greater than in the outpatients group (7.1 vs 40.8%, p=0.002) (Table 1).

## Inpatients: survivors vs. non-survivors

Comparing the baseline characteristics of survivor *versus* deceased patients with COVID-19 admitted to the hospital, the median age of non-survivors was significantly higher than the median age of survivors (65 vs. 57 years, p=0.047). No other differences were found between the two groups regarding demographics and previously known comorbidities. Black race was overrepresented among the non-survivors (30%) in comparison to survivors (7.7%), without reaching a significant difference (p=0.055) (Table 2).

Concerning clinical management and outcomes, ICU admission (40% vs 12.8%, p=0.048) and need for KRT (2.6 vs 50%, p<0.01) were more frequent among the patients who did not survive.

Additionally, treatment with remdesivir was significantly more frequent among patients who survived (46.2 vs. 10%, p=0.036) (Table 3).

**Table 3:** Clinical management and outcomes of hospitalized patients: survivors vs. non-survivors

	Inpatients (n=49)	Survivors (n=39)	Non-survivors (n=10)	*p*-value
ICU (%)	18.4 (n=9)	12.8 (n=5)	40 (n=4)	0.048
AKI (%)	49 (n=24)	46.2 (n=18)	60 (n=6)	0.435
KRT	12.2 (n=6)	2.6 (n=1)	50 (n=5)	<0.01
Immunosuppression adjustment (%)	95.9 (n=47)			
Anti-metabolite reduction or withdrawal	83.7 (n=41)	76.9 (n=30)	50 (n=5)	0.093
CNI reduction or withdrawal	12.2 (n=6)	15.4 (n=6)	10 (n=1)	0.664
mTORi reduction or withdrawal	14.3 (n=7)	10.3 (n=4)	10 (n=1)	0.981
Corticosteroid increase	81.6 (n=40)	71.8 (n=28)	60 (n=6)	0.470
Remdesivir (%)	38.8 (n=19)	46.2 (n=18)	10 (n=1)	0.036
Dexamethasone (%)	57.1 (n=28)	56.4 (n=22)	60 (n=6)	0.838

ICU: Intensive Care Unit; AKI: Acute Kidney Injury; KRT: Kidney Replacement Therapy; CNI: Calcineurin inhibitor; mTORi: mammalian target of rapamycin inhibitor.

## Discussion

The impact of COVID-19 in the general population has been very significant around the globe in the past year. At our center, SARS-CoV-2 infection was detected in approximately 8% of all kidney transplant recipients over a one-year period. Of these patients, the majority (63.6%) developed severe forms of the disease, requiring hospital admission. This percentage is consistent with the results of previous studies, where rates of hospitalization ranged from 68-78%^7^^-^ 9. Other series reported even greater rates of hospitalization particularly in patients with specific comorbidities, such as HIV infection^10^.^()^

Patient age was the only demographic feature that seemed to have impacted clinical outcomes in our cohort. In fact, hospitalized patients who did not survive were significantly older than the patients who survived. Globally, the median age of infected patients is in line with previous published case series^11^.

In our cohort, race did not significantly affect severity of disease or clinical outcomes, as pointed out in other series^9^^,^ 12. Nevertheless, a trend was noticed, as highlighted in the results. Even in those studies, worse outcomes associated to ethnical origin may reflect differences in underlying chronic conditions or difference in access to medical care units^12^^-^ 14, although increased expression of angiotensin-converting enzyme 2 (ACE2) receptor or lower vitamin D levels has been postulated as a potential biological justification for adverse outcomes^15^^,^ 16.

As showed in previous cohorts, comorbidities seem to impact disease severity and clinical outcomes in COVID-19. However, in our study, we did not find differences in comorbid diseases among hospitalized patients, when comparing survivors and non-survivors. Conversely, diabetes may have influenced severity of disease in this cohort, since there were significantly more diabetic patients who required hospitalization than among outpatients.

The optimal management of immunosuppression in transplant recipients with COVID-19 remains largely uncertain because of lack of objective data to support recommendations. To date, there is a general consensus in most transplant centers to reduce or discontinue antimetabolite therapy. However, the optimal management for calcineurin inhibitors is still unknown and different international recommendations on immunosuppression management have been published in 2020^17^^-^ 20. *In vitro* studies showed that non-immunosuppressive cyclosporin A derivatives effectively inhibit the expression of the N protein of the coronavirus, required for viral replication, making them potential candidates for the treatment of SARS-CoV-2 infection^21^. Additionally, tacrolimus was shown to inhibit viral replication of SARS-CoV at non-toxic, low-micromolar concentrations with a reduction in viral titers to undetectable levels^22^^,^ 23.

AKI occurred in almost half of hospitalized patients (49%, n=24) which is in concordance with results previously published in other centres^24^^,^ 25. In fact, AKI in kidney transplant recipients seems to occur more frequently compared to the general population, as pointed out by a French study that compared clinical outcomes in kidney transplant recipients to a single-center cohort of non-transplant patients. Greater susceptibility of kidney transplant recipients to dehydration, nephrotoxicity, and hemodynamic instability may explain the higher incidence of AKI^26^.

COVID-19 mortality rates among kidney transplant recipients have been reported to be higher than in the general population. However, these patients have comorbidities (such as hypertension, diabetes, cardiovascular disease, or renal dysfunction) that affect this outcome. In fact, multivariable analysis models have failed to show kidney transplantation as an independent risk factor for mortality^26^.

Interestingly, in our cohort, treatment with remdesivir was significantly more frequent among hospitalized patients who survived. Remdesivir is a nucleotide analogue initially developed to treat Ebola virus. It was not used until the outbreak of the COVID-19 pandemic, but has shown to be effective in the general population. Remdesivir has gained attention after the publication of studies including the randomized placebo-controlled ACTT1 trial, which showed a statistically significantly shorter time to recovery^27^^,^ 28. There are relatively few published data on the safety and efficacy of remdesivir in solid organ transplant recipients. However, given the potential benefit, transplant clinicians are encouraged to consult pharmacy specialists at their center and consider the possible use of remdesivir in patients with impaired kidney function^29^.

Our study has several limitations, as the analyzed data represent a heterogeneous cohort of patients with different demographic, comorbidities, and immunological characteristics. The small number of patients and outcomes precludes a robust multivariate analysis. Another potential limitation of the study is that asymptomatic patients or patients with very mild symptoms may not have reported the disease to the KTC, potentially affecting the percentage of ambulatory patients versus patients requiring admission. Although data collection was based in electronic and paper medical records, clinical outcomes such as AKI or immunosuppression changes could have been underreported, influencing the results. Additionally, we did not compare kidney transplant recipients with age- and comorbidity-matched general population, so we cannot be precise about the extent to which kidney transplantation and immunosuppression status impacted COVID-19 outcomes. Other studies are consistent with this limitation^26^.

## Conclusion

We present data from a single-center cohort of kidney transplant recipients with SARS-CoV-2 infection, including clinical data of outpatients and hospitalized patients. For hospitalized patients, immunosuppression management included reduction or withdrawal of antimetabolite and increasing of corticosteroid dose. AKI was highly frequent in our cohort, reaching almost half of hospitalized patients. Global mortality (among outpatients and inpatients) was 13%, but among hospitalized patients mortality reached 20%, reflecting greater severity of the disease.
